# Applications of digital PCR in COVID‐19 pandemic

**DOI:** 10.1002/VIW.20200082

**Published:** 2021-01-01

**Authors:** Chianru Tan, Dongdong Fan, Nan Wang, Fang Wang, Bo Wang, Lingxiang Zhu, Yong Guo

**Affiliations:** ^1^ Department of Biomedical Engineering, School of Medicine Tsinghua University Beijing China; ^2^ TargetingOne Corporation Beijing China; ^3^ National Research Institute for Family Planning Beijing China

**Keywords:** COVID‐19, digital PCR, nucleic acid testing, SARS‐CoV‐2

## Abstract

The coronavirus disease 2019 (COVID‐19) pandemic has led to a public health crisis and global panic. This infectious disease is caused by a novel coronavirus named severe acute respiratory syndrome coronavirus 2 (SARS‐CoV‐2). Digital polymerase chain reaction (dPCR), which is an emerging nucleic acid amplification technology that allows absolute quantification of nucleic acids, plays an important role in the detection of SARS‐CoV‐2. In this review, we introduce the principle and advantages of dPCR, and review the applications of dPCR in the COVID‐19 pandemic, including detection of low copy number viruses, measurement of the viral load, preparation of reference materials, monitoring of virus concentration in the environment, detection of viral mutations, and evaluation of anti‐SARS‐CoV‐2 drugs. We also discuss the challenges of dPCR in clinical practice.

## Graphical abstract

The coronavirus disease 2019 (COVID‐19) pandemic has led to a public health crisis and global panic. Digital polymerase chain reaction (dPCR), which is an emerging nucleic acid amplification technology that allows absolute quantification of nucleic acids, plays an important role in the detection of the pathogen of COVID‐19. In this review, the authors introduce the principle and advantages of dPCR, review the applications of dPCR in the COVID‐19 pandemic, and discuss the challenges of dPCR in clinical practice.

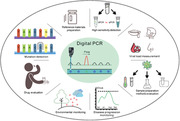

## 1 INTRODUCTION

A pandemic caused by severe acute respiratory syndrome coronavirus 2 (SARS‐CoV‐2) broke out in December, 2019.^1^ The pathogen SARS‐CoV‐2, same as MERS‐CoV, SARS‐like bat CoV, and SARS‐CoV, belongs to the betacoronavirus clade, and genome analysis found that SARS‐CoV‐2 is the closest to the SARS‐like bat CoV.^2^ The infectious disease caused by this pathogen was named coronavirus disease 2019 (COVID‐19). The COVID‐19 pandemic has led to a public health crisis and global panic. By 10 November 2020, over 50.9 million cases of COVID‐19 have been reported, including 1.2 million deaths (https://coronavirus.jhu.edu/map.html).

COVID‐19 has strong infectiousness, so it is important to diagnose the disease timely and correctly for treatment and epidemic control. Viral nucleic acid testing is the main method for diagnosing COVID‐19, and reverse transcriptase quantitative polymerase chain reaction (RT‐qPCR) is the most widely employed technology in the clinic.^3^ Some other techniques have also been developed for SARS‐CoV‐2 RNA detection, including digital PCR (dPCR), reverse transcription loop‐mediated isothermal amplification (RT‐LAMP), and transcription‐mediated amplification (TMA).^4^ dPCR is an emerging nucleic acid amplification technology that allows absolute quantification of nucleic acids. It provides such advantages over qPCR as higher sensitivity, higher precision, and higher resistance to inhibitors. Hence, dPCR‐based assays for detection of SARS‐CoV‐2 have been developed to facilitate the diagnosis and related researches of COVID‐19.

Here, we introduce the principle and advantages of dPCR, review the applications of dPCR in COVID‐19 pandemic, and discuss the challenges of dPCR in COVID‐19 pandemic and relevant clinical applications.

## 2 DIGITAL PCR TECHNOLOGY

### 2.1 Principle of digital PCR

PCR is a molecular technique for exponential amplification of a specific segment of DNA, which was developed by Kary Mullis in the 1980s.^5^ The concept of “dPCR” was first mentioned by Vogelstein and Kinzler in 1999.^6^ The principle of dPCR is to partition the traditional PCR reaction mixture into many independent subreactions before amplification, and to determine the original number of target molecules via counting the partitions showing positive and negative PCR results and analyzing with the Poisson distribution after amplification. Generally speaking, dPCR could be classified into two types: droplet‐based dPCR and chip‐based dPCR. Droplet‐based dPCR partitions the PCR reaction mixture by microfluidic droplet technique.^7^ Chip‐based dPCR achieves the partition using a base plate that is equipped with tens of thousands of microwells by micro‐/nanofabrication techniques.^8^

**FIGURE 1:**
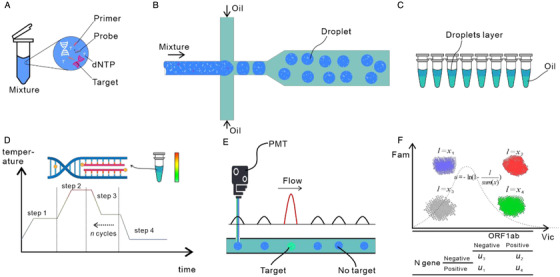
Droplet digital PCR workflow. (A) A mixture containing dNTPs, primers, and probes is prepared for the amplification reaction. (B) Water‐in‐oil droplets are generated using a microfluidic flow‐focusing system. (C) The generated droplets and oil are collected in PCR tubes. (D) The PCR tubes are placed in a thermal cycler; PCR amplification occurs in each droplet. (E) After amplification, the droplets are checked by a photoelectric detection system composed of lasers and photomultiplier tubes. The fluorescence of droplets containing target molecules is strong enough to be distinguished from the background fluorescence. (F) Finally, the fraction of positive droplets is fitted to the Poisson distribution to determine the absolute copy number of target molecules in the original reaction mixture

Taking the SARS‐CoV‐2 detection based on droplet‐based dPCR as an example, we show the workflow of dPCR in Figure 1. First, a PCR reaction mixture including target primers and fluorescent markers is prepared (Figure 1A), and generally, the fluorescent markers are either Taqman probes or a fluorescent DNA binding dye. Typically, the Taqman technique is used in SARS‐CoV‐2 detection, and the nucleocapsid gene and orf1ab gene are chosen as the targets. Then, water‐in‐oil emulsion droplets are generated using a microfluidic chip with crossing channels (Figure 1B). The generated droplets with a diameter between 90 and 120 μm may contain zero, one, or multiple target molecules. The distribution of the targets in the partitions follows a Poisson distribution. The droplets are subsequently heated by a thermal cycler for amplification (Figure 1D). After thermal cycling, the number of the target molecules in droplets that contain one or more of the molecules increases to tens of billions, so the fluorescent signal in the droplets can be detected. Next, the droplets are checked by a photoelectric detection system composed of lasers and photomultiplier tubes. The fluorescence of droplets containing target molecules is strong enough to be distinguished from the background fluorescence (Figure 1E). According to the signal amplitude, each droplet is classified as positive or negative. Using the Poisson distribution, the fraction of positive droplets is calculated to determine the absolute copy number of target molecules in the original reaction mixture. Figure 1F illustrates the analysis results of a dual‐target dPCR reaction, where the clusters in the 2D view have different combinations of targets.

### 2.2 Advantages of digital PCR

*Absolute quantification*: In qPCR, the fluorescence of the amplified target molecules is monitored after each amplification cycle. The copy number of target molecules is quantified by mapping the relation between the cycle threshold (*Ct*) of the fluorescence amplification curve with a standard curve generated from a reference material. The amplification curves are usually vulnerable to inadequate amplification efficiency and sample quality, inhibitors, and system error. In contrast, dPCR divides the PCR reaction mixture into tens of thousands or even millions of reaction units, and the fluorescence of each unit is detected at the end of amplification. The copy number of target molecules is calculated by the fraction of the reaction units containing the target molecules, according to the Poisson distribution. Consequently, dPCR is less sensitive to inhibitors than qPCR, and copy number calculation does not rely on reference materials. Therefore, dPCR is eligible to provide more reliable and accurate quantification results than qPCR. For instance, Hindson et al.^9^ compared the ability of dPCR and qPCR to quantify miRNAs, and the results indicated that dPCR indeed offered more accurate results.

*Higher sensitivity*: As aforementioned, dPCR partitions the reaction mixture into tens of thousands of subreactions. Therefore, the number of nontarget DNA is decreased by a few orders of magnitude in each subreaction that contains the targets (e.g., when the PCR is discretized into 10,000 reaction units, the nontarget DNAs in each unit is reduced by 4 orders of magnitude). As a result, the nonspecific interference exerted on targets decreases by several orders of magnitude, which benefits the valid amplification of the targets. Hence, dPCR is equipped with high sensitivity and is able to precisely quantify rare targets. For example, in the detection of rare BRAF V600E mutation, dPCR and qPCR could detect 0.001% and 1% mutant fraction, respectively.^7^

*Better stability*: Discretization also separates the amplification inhibitors in the reaction mixture into many individual reaction units, which reduces the total number of inhibitors in each unit and thus improves the resistance to inhibitors. In the detection of human cytomegalovirus, dPCR showed better inhibitor tolerance than qPCR. Moreover, even though inhibitors may influence PCR amplification in a dPCR assay, the affected subreactions can still be correctly identified because their fluorescence intensity is still significantly higher than background fluorescence.^10^, ^11^

*Higher precision*: qPCR can routinely resolve a twofold difference in copy number in a single assay because the template doubles at each cycle and the fluorescence intensity is twofold higher after a cycle. In dPCR, it is theoretically possible to detect a difference in one target molecule through discretizing the PCR reaction mixture, and increasing the number of the partitions can improve the precision. For example, in a study of quantitative detection of human cytomegalovirus, the coefficient of variation (CV) of dPCR was lower than qPCR: the CV was fourfold and 1.5‐fold lower for dPCR compared with qPCR at the viral copy number of 10,000 copies/mL and 1000 copies/mL, respectively.^12^

## 3 APPLICATIONS OF DIGITAL PCR IN COVID‐19 PANDEMIC

### 3.1 High‐sensitivity detection of SARS‐CoV‐2

Although RT‐qPCR is currently the gold standard for the diagnosis of COVID‐19, a high false‐negative rate of RT‐qPCR tests for SARS‐CoV‐2 has been reported.^13^, ^14^ Several factors may be related to the false‐negative results, including mutations in the primer‐ and probe‐targeted regions, insufficient quantities of the virus in the sample due to inappropriate collection or handling, and the presence of amplification inhibitors in the sample.^15^ As dPCR offers higher sensitivity and higher tolerance to inhibitors, it has been used to detect SARS‐CoV‐2 to reduce the incidence of false‐negative results. Comparison of the limit of detection (LoD) between dPCR and RT‐qPCR detection assays for SARS‐CoV‐2 showed that the dPCR assay has lower LoD.^16^, ^17^, ^18^ Moreover, dPCR showed higher sensitivity than RT‐qPCR in the testing of clinical samples. Some samples from patients with confirmed COVID‐19 were classified as positive by dPCR but found inconclusive or negative by RT‐qPCR.^16^, ^18^, ^19^, ^20^, ^21^, ^22^ For example, Suo et al.^16^ showed that 26 samples from COVID‐19 outpatients with negative RT‐qPCR test results were positively identified by dPCR. In addition, Falzone et al.^17^ found that dPCR was less sensitive to the interference of amplification inhibitors. These results indicate that dPCR is superior to RT‐qPCR in the detection of low‐viral load samples and can be used as a complement to RT‐qPCR.

### 3.2 Evaluation of the viral load in different types of clinical samples

Many types of clinical samples can be used in COVID‐19 testing, including nasal swabs, throat swabs, sputum, blood, urine, stool, and bronchoalveolar lavage fluid. Selecting an appropriate type of sample is crucial for accurate clinical diagnosis. To increase the positive detection rate of COVID‐19 testing, it is necessary to evaluate the viral load of each type of sample and choose the sample type with the highest viral load when possible. As dPCR allows absolute quantification, it plays an important role in the quantification of viral loads in clinical samples of COVID‐19 patients. For example, Yu et al.^19^ used dPCR to quantify the viral load in nasal swabs, throat swabs, sputum, blood, and urine. They found that sputum had the highest viral load, followed by nasal swabs and throat swabs. The results suggested that using sputum as a test sample may increase the positive detection rate.

### 3.3 Evaluation of sample preparation methods

Safe handling of samples from suspected COVID‐19 cases is critical to protect medical laboratory personnel from infection. Inactivation of the virus is performed by most laboratories to ensure that the samples are safe to handle at a reduced biosafety level. However, the inactivation treatment may affect the quality of the sample and lead to false‐negative results. Therefore, it is necessary to understand the specific impact of sample pre‐treatment on the test results. Chen et al.^23^ used dPCR to quantify the viral load in samples before and after three inactivation methods. The results indicated that the inactivation treatment reduced the quantity of detectable viral RNA, which may lead to false‐negative results. The authors found that the viral load in the heat‐inactivated sample was greatly reduced, and the use of the TRIzol reagent had the least effect on the quantity of viral RNA. This study shows that dPCR is a powerful tool for evaluating the influence of pre‐treatment on nucleic acid samples.

### 3.4 Dynamic monitoring of disease progression

In clinical management and treatment, it is necessary to monitor the progress of the patient's illness. For most viruses, a higher viral load is associated with more severe illness. Therefore, it is important to study the relationship between viral load and the severity of COVID‐19. The high sensitivity and high precision of dPCR make it capable of monitoring changes in the viral load in patients. Yu et al.^19^ used dPCR to quantify the viral load in samples from patients at different time points throughout their course of disease. They found that the viral load in the early and progressive stages was significantly higher than that in the recovery stage, suggesting that dynamic monitoring of the viral load helps to assess the progression of the disease.

### 3.5 Preparation of nucleic acid reference materials

Nucleic acid reference materials are used to evaluate the performance of detection methods and construct standard curves for RT‐qPCR. Reference materials also serve as a positive control for the detection of SARS‐CoV‐2 nucleic acid. dPCR enables absolute quantification of nucleic acids, thus it has been used to characterize nucleic acid reference materials. For example, van Kasteren et al.^24^ used dPCR to quantify the copy number of SARS‐CoV‐2 for the preparation of reference materials. They further used serial dilutions of viral RNA to evaluate the analytical performance of different RT‐qPCR kits. Fung et al.^25^ quantified viral nucleic acid in a positive patient material by dPCR and then prepared serial dilutions to determine the analytical limits of seven SARS‐CoV‐2 detection methods. dPCR is essential for national institutions such as the National Institutes for Food and Drug Control of China and the National Institute of Metrology of China or private enterprises engaged in the development of SARS‐CoV‐2 testing methods to prepare SARS‐CoV‐2 nucleic acid reference materials.

### 3.6 Monitoring the virus concentration in the environment

Understanding the modes of transmission of SARS‐CoV‐2 and its distribution in the environment are critical to the formulation of disease prevention and control strategies. A detection method with high sensitivity and high tolerance to inhibitors is needed to accurately test samples collected in diverse environments. Liu et al.^26^ used dPCR to measure viral RNA in aerosols in different areas of two hospitals in Wuhan during the COVID‐19 outbreak. They found that airborne SARS‐CoV‐2 RNA was undetectable in most public areas. The concentration of viral RNA in aerosols was low in isolation wards and ventilated wards, but it was high in the toilets used by the patients and certain medical staff areas. According to these results, they suggested that SARS‐CoV‐2 may have the potential to be spread through aerosols. Lv et al.^27^ used RT‐qPCR and dPCR to detect virus remaining on the surface of laboratory‐related objects such as sample transportation and reception‐related facilities, testing instruments, and personal protective equipment. All samples were found negative by RT‐qPCR, while 13 of 61 samples were considered positive by dPCR. The areas with the highest density of SARS‐CoV‐2 RNA were the outer gloves of laboratory personnel handling viral samples. Moreover, the other objects with positive results were directly or indirectly touched by those gloved hands, indicating that hand contact is the main route of transmission. These studies demonstrated that dPCR can detect and measure viral RNA in diverse environments, providing a reference for disease prevention strategies and disinfection procedures.

### 3.7 Detection of SARS‐CoV‐2 mutations

RNA viruses have high mutation rates, which are correlated with virulence and evolvability. Viral mutation research is essential for evaluating viral drug resistance and understanding the mechanisms related to immune escape and pathogenesis. Viral mutations can be identified by next‐generation sequencing, but the detection of known genetic mutations in laboratory samples and clinical samples requires a more sensitive technology. Wong et al.^28^ developed a dPCR‐based assay to detect SARS‐CoV‐2 with bat‐like PRRA deletion (SARS‐CoV‐2_△PRRA_) in Vero‐E6‐propagated isolates, human organoids, experimentally infected hamsters, and COVID‐19 patients. They found that SARS‐CoV‐2_△PRRA_ naturally existed in COVID‐19 patients and was transmissible. However, these variants only appeared with very low frequency in COVID‐19 patients, indicating that the wild type with the PRRA insertion was more competitive than SARS‐CoV‐2_△PRRA_. This study demonstrated that dPCR is suitable for the detection of known SARS‐CoV‐2 mutations, especially for some low‐frequency mutations.

### 3.8 Evaluation of anti‐SARS‐CoV‐2 drugs

A reduction in the viral load is an important indicator of the effectiveness of antiviral drugs. For example, in a study of remdesivir use in adult patients with severe COVID‐19, Wang et al.^29^ monitored the viral load in the subjects’ respiratory tract specimens to determine whether the drug could accelerate the decline in the viral load or reduce the virus detection rate. Furthermore, in a comparative study of arbidol and lopinavir/ritonavir in treating COVID‐19 patients, Zhu et al.^30^ measured changes in the viral load to evaluate the efficacy of the therapy. Both of these studies used RT‐qPCR to measure changes in the viral load. Wang et al. used a standard curve method to determine the viral load, while Zhu et al. used the *Ct* value to indicate the change in the viral load. Since dPCR allows quantification without a standard curve, and it is highly precise, the authors believe that dPCR is more suitable for evaluating the effectiveness of anti‐SARS‐CoV‐2 drugs than RT‐qPCR.

## 4 SUMMARY AND PERSPECTIVE

As an emerging technology for absolute quantification of target nucleic acids, dPCR is highly sensitive and specific to low‐abundance DNA and highly resistant to amplification inhibitors. Thus, dPCR can be used for detection of low copy number viruses, measurement of the viral load, preparation of reference materials, monitoring of virus concentration in the environment, detection of viral mutations, and evaluation of anti‐SARS‐CoV‐2 drugs.

Although the advantages and clinical application prospects of dPCR have been demonstrated in many studies, some challenges need to be overcome so that dPCR can be widely used in different types of medical units, including primary hospitals and epidemic areas: (1) the sample throughput of the available dPCR systems needs to be improved to fully meet the requirement of mass screening for COVID‐19; (2) a highly automated digital PCR system needs to be developed to simplify the operation process of dPCR and reduce hands‐on time; (3) the cost of dPCR instruments and reagents needs to be reduced; (4) clinical laboratory standards and guidelines of dPCR are required to assure the quality of the results; (5) multicenter evaluation is required to test the performance of dPCR‐based SARS‐CoV‐2 nucleic acid detection kits, and the detection kits need to be approved by regulatory agencies such as China's National Medical Products Administration (NMPA) and the United States Food and Drug Administration (FDA). With the joint efforts of researchers and the industry, dPCR can be widely used in laboratories or hospitals in the near future to assist scientific research and solve clinical problems.

## CONFLICT OF INTEREST

The authors declare no conflict of interest.
